# A *Borrelia burgdorferi* outer surface protein C (OspC) genotyping method using Luminex technology

**DOI:** 10.1371/journal.pone.0269266

**Published:** 2022-06-01

**Authors:** Patrick Pearson, Olivia Skaltsis, Chu-Yuan Luo, Guang Xu, Zachary Oppler, Dustin Brisson, Stephen M. Rich

**Affiliations:** 1 Laboratory of Medical Zoology, Department of Microbiology, University of Massachusetts, Amherst, Massachusetts, United States of America; 2 NH Citizens Health Initiative, Institute for Health Policy and Practice, University of New Hampshire, Concord, New Hampshire, United States of America; 3 Evolution and Ecology Disease Systems Laboratory, Department of Biology, University of Pennsylvania, Philadelphia, Pennsylvania, United States of America; University of Kentucky College of Medicine, UNITED STATES

## Abstract

*Borrelia burgdorferi* is an important tickborne human pathogen comprising several strains based on nucleotide sequence of the outer surface protein C (*ospC*) gene. Detection and characterization of different *ospC* genotypes is vital for research on *B*. *burgdorferi* and the risk it poses to humans. Here we present a novel, multiplex assay based on Luminex xMAP technology for the detection of *B*. *burgdorferi ospC* genotypes. The assay has five major steps: amplification of the *ospC* gene, hydrolyzation of surplus primers and nucleotides, incorporation of biotinylated nucleotides into the template DNA, hybridization to Luminex microspheres, and detection of fluorescent signals corresponding to each *ospC* genotype. We validated the protocol by comparing results obtained from our method against results from an established *ospC* genotyping method. This protocol can be used for the characterization of *ospC* genotypes in *B*. *burgdorferi* infected ticks, reservoir hosts, and/or clinical samples.

## Introduction

*Borrelia burgdorferi sensu stricto* (hereafter *B*. *burgdorferi*) is the tick-borne bacterium that causes human Lyme disease in the United States [1]. The genetic locus that encodes the outer surface protein C (OspC) protein is highly variable, with at least 19 major alleles circulating among reservoir host and tick (*Ixodes scapularis*) populations in the northeastern United States [2–5]. An *ospC* allele group, or genotype, is composed of members with less than 2% nucleotide sequence difference within a genotype and more than 8% nucleotide sequence difference between genotypes [2, 4]. While molecular detection of *B*. *burgdorferi* in host and tick samples is commonplace, *ospC* genotyping of those *B*. *burgdorferi* samples is seldom done. This has potential public health significance since there is evidence suggesting that only a subset of *ospC* genotypes (A, B, I, K, and N) cause the overwhelming majority of disseminated infections in humans [6–8]. Identifying the *B*. *burgdorferi ospC* genotypes in natural tick and host populations, and determining which *ospC* genotypes routinely infect humans, can improve research on the basic biology of this pathogen and its associated public health risks.

An established method to detect different *ospC* genotypes is the reverse line blotting (RLB) assay [3–5, 9, 10]. This DNA-DNA hybridization method of RLB is a robust protocol for detecting *ospC* genotypes. However, RLB cannot readily be adapted to a high-throughput process. Recently, a method based on next generation sequencing (NGS) was developed for *ospC* genotyping [11]. The NGS method is an improvement over RLB, but also requires a bioinformatics pipeline. To overcome these shortcomings, we developed an *ospC* genotyping protocol using Luminex multiplex technology in a 96-well plate format that is straightforward, provides same-day results, and offers improved efficiency compared to RLB (S1 Table).

Luminex xMAP technology is powered by spectrally distinct MagPlex-TAG microspheres (beads) that are coupled with a unique 24 bp oligonucleotide sequence (anti-TAG) [12]. The coupled beads allow the end user to build a custom assay by designing primers that are specific for their target and modified with a 5’ oligonucleotide sequence (TAG) that is complementary to the anti-TAG sequence on the matched bead set. MAGPIX is a fluidics-based instrument equipped with a CCD camera capable of detecting up to 50 analytes in a single well. The CCD camera records presence of beads based on emissions from internal dyes. Each anti-TAG/bead combination has a unique fluorescent signature allowing for detection of a corresponding sequence-specific analyte, which in the case of our protocol correspond to individual *ospC* nucleotide sequences.

The *ospC-*specific probes previously used in the RLB method for DNA hybridization [3–5] were repurposed, with some minor modifications, as primers in this assay by adding the 5’ TAG sequence described above. Each *ospC* primer is paired with a corresponding bead type. The step-by-step protocol is described in the S1 File. Briefly, *ospC* genotypes are amplified via nested PCR (S2 Table) and the product is enzymatically purified. In the allele-specific primer extension (ASPE) step, primers bind to their *ospC* target, and biotinylated nucleotides are incorporated during extension. The biotin-labelled product is then hybridized to the complementary beads during a subsequent incubation step. A fluorescently labelled streptavidin solution is added, and the samples are analyzed on the MAGPIX instrument. In each well a median fluorescent intensity (MFI) value is generated for each *ospC* genotype.

The assay was designed using general recommendations from the Luminex xMAP Cookbook [12] with subsequent optimization. The original K probe, as designed by Qiu et al. [3], was discovered to have a 4 base pair mismatch with the *ospC* K genotype sequence (S2 File). The K primer was redesigned and increased the MFI signal when detecting the *ospC* K genotype compared to the original K primer (S1 Fig). The optimized and validated Luminex *ospC* genotyping (LOG) assay is relatively simple, scalable, and efficient. It is appropriate for studies that require identification of *ospC* genotypes in *B*. *burgdorferi* positive samples. Due to the multiplex platform, the protocol can be modified as needed to include additional analytes for detection of yet to be discovered, novel *ospC* gene sequences.

The nucleotide sequence of *ospC* genotype C is a chimera of other genotypes. We were not able to identify a primer/probe sequence to distinguish the chimera from the donor sequences. This is a known shortcoming of hybridization based genotyping approaches, including RLB [3, 4]. As is the case with RLB, the E/C primer hybridizes with both genotype E and C nucleotide sequences, while the I/C primer hybridizes with both genotype I and C nucleotide sequences [4]. Therefore, it is not possible to unambiguously detect the presence of genotype C in samples that are multiply infected with genotypes E and I. In contrast to NGS based approaches, our protocol only detects known *ospC* genotypes for which there is a specific primer. However, the presence of novel *ospC* genotypes can be inferred where an amplicon hybridizes with the “ALL” primer but fails to hybridize with any of the known nucleotide sequences (S3 File). In practice, these novel genotypes can then be characterized by PCR and Sanger sequencing.

## Materials and methods

The protocol described in this peer-reviewed article is published on protocols.io, dx.doi.org/10.17504/protocols.io.b4t8qwrw and is included for printing as the S1 File.

All statistical analyses were performed using GraphPad Prism version 9.0. A p-value of < 0.05 was considered significant and a Bonferroni correction was used to account for multiple comparisons. The data from the validation experiment is available in the S4 File.

## Expected results

To validate the LOG assay against a biologically relevant sample set, *B*. *burgdorferi ospC* DNA was amplified from adult, field-collected, *I*. *scapularis* ticks (N = 152) and analyzed using the developed assay. The *ospC* genotypes infecting the ticks used in the present study had previously been determined using the RLB method [5]. Therefore, this experiment allowed us to simultaneously verify that LOG can detect the 19 *ospC* genotypes and directly compare results between this assay and RLB.

The LOG assay detected multiple *ospC* infections, including the identification of 12 different *ospC* genotypes in a single tick sample. The average number of *ospC* genotypes per tick was 3.51 and 3.30 for LOG and RLB, respectively.

To compare the genotypes detected in each sample by both methods, 152 pairwise comparisons were performed (Table 1). Genotype C was excluded during these analyses since the RLB method previously used to analyze the samples did not have specific probes for this genotype. Of the 152 comparisons, 65 (42.8%) had identical results from the two methods and 36 (23.7%) showed RLB genotypes as a subset of those detected by LOG. For 37 samples (24.3%), the genotypes detected by LOG were a subset of those detected by RLB. For 14 samples (9.2%) each method detected at least one genotype that was absent from the results of the other method.

**Table 1:** Pairwise comparison results for each sample using LOG and RLB (N = 152 samples).

Outcome	N (%)
Identical results	65 (42.8%)
RLB genotypes a subset of LOG genotypes	36 (23.7%)
LOG genotypes a subset of RLB genotypes	37 (24.3%)
Each method detected ≥ 1 genotype absent from results of the other method	14 (9.2%)
Total	152 (100%)

**Fig 1:**
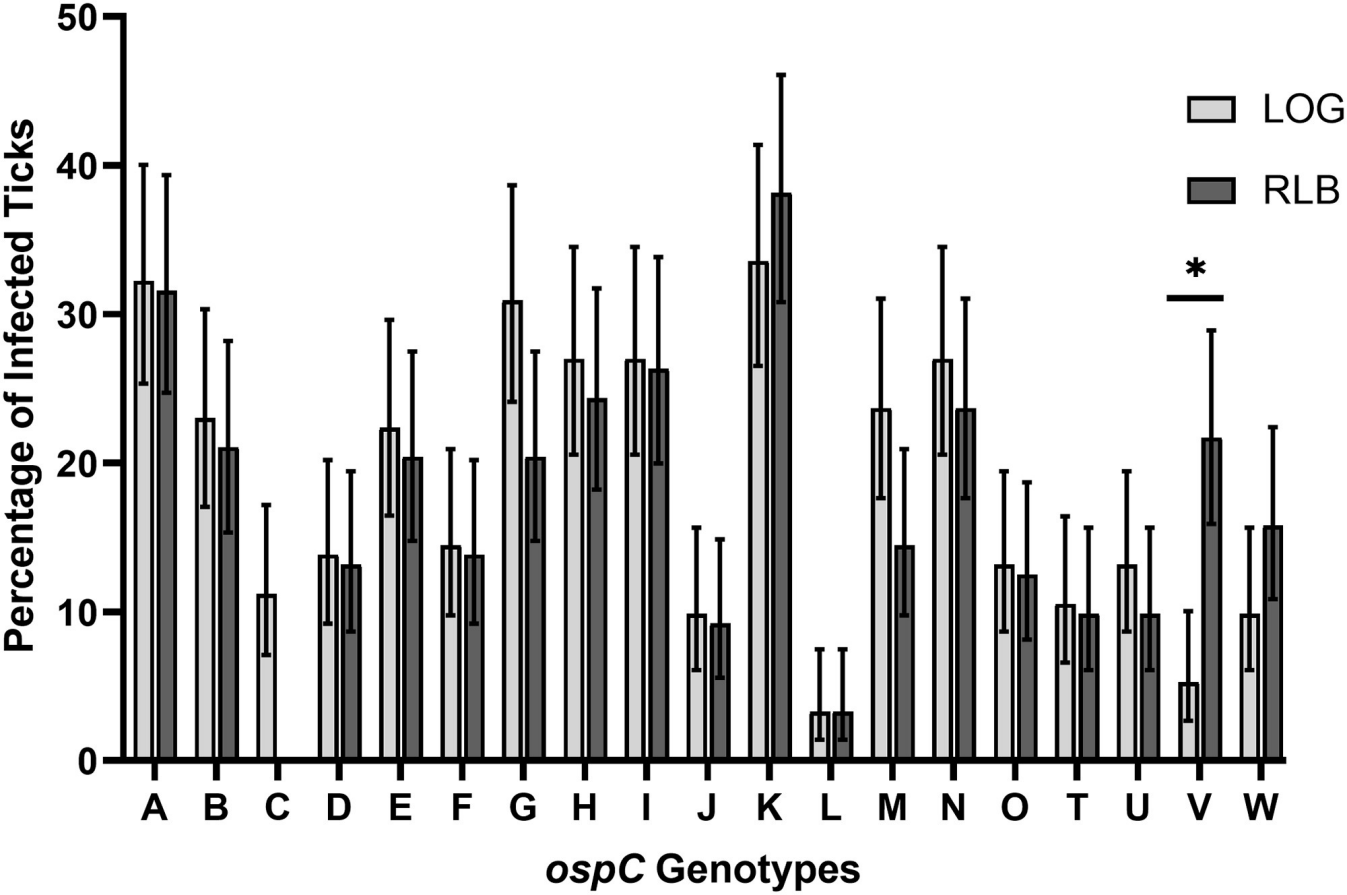
*ospC* genotype detection frequencies using LOG and RLB. Error bars represent 95% confidence intervals of the proportion. The detection frequency of genotype V was significantly different between methods (Fisher’s exact test, P<0.0001).

All 19 genotypes for which we have specifically designed primers were detected using the LOG assay and at relatively similar frequencies compared to RLB (Fig 1). It appears that *ospC* genotypes G and M are overrepresented, and that *ospC* genotypes V and W are underrepresented by LOG. However, after correcting for multiple comparisons, only *ospC* genotype V was detected at significantly different frequencies between the two methods (Fisher’s exact test, P<0.0001). One explanation could be that *ospC* genotype V is not being properly detected by our new method. However, during the optimization process using synthetic double-stranded *ospC* gene fragments (gBlocks, IDT), *ospC* genotype V was routinely detected in single and mixed infections (S3 Table). Another explanation could be that the *ospC* V probe was hybridizing with off-target templates in RLB. Based on the pairwise comparison analysis, there were 29 samples positive for an *ospC* V genotype according to RLB, but negative according to LOG. Of those, nearly all (28/29) were also coinfected with *ospC* genotypes F, M, or O as confirmed by both methods. Utilizing the RLB results from all 152 samples, a positive association was found between genotypes V and F (Fisher’s exact test, P<0.0001; odds ratio 21.46, 95% CI 6.89–56.98) and genotypes V and O (Fisher’s exact test, P<0.0001; odds ratio 9.143, 95% CI 3.1 to 23.33). Since the V probe used in RLB shares the same sequence with the V primer in LOG, we suspect that cross-reactivity of the V probe with other genotypes may occur during DNA hybridization in RLB but is less likely to be detected in our developed assay that requires hybridization and extension of the target to generate a positive signal. It is also important to consider that every sample went through two separate nested PCR reactions: one for RLB and one for LOG. Therefore, any differences between methods may be attributable to DNA degradation by repeated freeze/thaw cycles and different nested PCR efficiencies.

The LOG assay performed comparably well using *B*. *burgdorferi* infected tick samples that had been previously genotyped by RLB. The new *ospC* genotyping protocol can detect multiple *ospC* infections and most of the pairwise results (101/152, 66.4%) were either an exact match or additional genotypes were detected by LOG compared to RLB. All 19 genotypes designed for our assay were detected and more genotypes can be added as needed going forward. The LOG assay has demonstrated utility and offers another tool for researching *B*. *burgdorferi*.

## Supporting information

S1 File *Borrelia burgdorferi ospC* genotyping using Luminex technology, also available on protocols.io. (PDF)

S2 File Comparison of the Qiu et al. K probe and modified K primer used in this assay. The Qiu et al. K probe and our modified K primer sequences are shown in Table 1, nucleotide differences are highlighted in bold. Fig 1 shows an alignment of the Qiu et al. K probe and our modified K primer with an *ospC* K reference sequence (Genbank accession number AY275214).
(DOCX)

S3 File Alignment of *ospC* reference sequences showing nested PCR and ASPE primer hybridization sites. The nested PCR first round primer hybridization sites are highlighted in blue and the second round primer hybridization sites are highlighted in red. Overlapping regions for the first and second round forward primers are highlighted in purple. The hybridization site for the ASPE “ALL” primer is highlighted in green. The ASPE primer hybridization sites for each *ospC* genotype are highlighted in yellow. The *ospC* A-U reference sequences were obtained from Di et al. [11]. The *ospC* V and W accession numbers are FJ649656 and FJ649657, respectively.
(DOCX)

S4 File *ospC* genotyping results from tick samples (N = 152) using LOG and RLB. (XLSX)

S1 Fig Optimization of the K primer. *ospC* genotype K was amplified from a plasmid stock and diluted to approximately 7 ng/ul. Duplicate samples of the genotype were detected using two ASPE primer mixes: one with the original Qiu et al. K primer and the other with the modified K primer. Average MFI values with standard deviation as the error bars are shown for each treatment.
(DOCX)

S1 Table Time efficiency comparison between LOG and RLB. For LOG, a representative run of 90 samples was chosen to leave room on the 96-well plate for negative and positive controls, as detailed in the step-by-step protocol.
(DOCX)

S2 Table Nested PCR *ospC* primers. (DOCX)

S3 Table Testing of single and mixed infections. *ospC* genotype standards (*ospC* gBlocks and a K plasmid stock) were amplified, diluted to approximately 5 ng/ul in samples representing single and mixed *B*. *burgdorferi ospC* genotype infections, and then analyzed using LOG. As described in the step-by-step protocol, a ratio to NTC value of ≥ 3 was considered positive.
(DOCX)
