# Supplementary material for: A Borrelia burgdorferi outer surface protein C (OspC) genotyping method using Luminex technology
Source: PLoS One. 2022 Jun 1;17(6):e0269266. doi: 10.1371/journal.pone.0269266 (PMC9159548; doi:10.1371/journal.pone.0269266)
Supplement: S1 File — (PDF) [file pone.0269266.s001.pdf]

Borrelia burgdorferi ospC Genotyping Using Luminex Technology

Patrick Pearson[1], Olivia Skaltsis[2], Chu-Yuan Luo[1], Guang Xu[1], Zachary Oppler[3], Dustin Brisson[3], Stephen M Rich[1]

[1]Laboratory of Medical Zoology, Department of Microbiology, University of Massachusetts, Amherst, Massachusetts, United States of America;

[2]NH Citizens Health Initiative, Institute for Health Policy and Practice, University of New Hampshire, Concord, New Hampshire, United States of America;

[3]Evolution and Ecology Disease Systems Laboratory, Department of Biology, University of Pennsylvania, Philadelphia, Pennsylvania, United States of America

dx.doi.org/10.17504/protocols.io.b4t8qwrw

Patrick Pearson

Borrelia burgdorferi is an important tickborne human pathogen and can be grouped into separate strains based on the outer surface protein C (*ospC*) gene. The detection and characterization of different *ospC* genotypes is vital for research on *B. burgdorferi* and the risk it poses to humans. Here we present a novel, multiplex assay based on Luminex xMAP technology for the detection of *B. burgdorferi ospC* genotypes. The assay has 5 major steps: amplification of the *ospC* gene, enzymatic purification, incorporation of biotinylated nucleotides into the template DNA, hybridization to Luminex microspheres, and detection of fluorescent signals corresponding to each *ospC* genotype. This protocol can be used for the characterization of *ospC* genotypes in *B. burgdorferi* infected ticks, reservoir hosts, and/or clinical samples.

DOI

dx.doi.org/10.17504/protocols.io.b4t8qwrw

Patrick Pearson, Olivia Skaltsis, Chu-Yuan Luo, Guang Xu, Zachary Oppler, Dustin Brisson, Stephen M Rich . Borrelia burgdorferi ospC Genotyping Using Luminex Technology. **protocols.io**
https://protocols.io/view/borrelia-burgdorferi-ospc-genotyping-using-luminex-b4t8qwrw

Borrelia burgdorferi, ospC, genotyping

protocol ,

Feb 08, 2022

Feb 22, 2022

57952

The Luminex xMAP Cookbook is available on the Luminex website and helpful for designing and troubleshooting genotyping assays.

The nucleic acid extraction depends on the type of sample being tested. We recommend using MasterPure™ Complete DNA and RNA Purification Kit (Lucigen) and following their protocols.

Positive controls are heavily recommended. One approach to generate positive controls is to use synthetic, double-stranded gene fragments (gBlocks) from IDT, corresponding to each *ospC* genotype. Amplify the gBlocks and mix into pools of positive controls accounting for every *ospC* genotype.

Reagents and Consumables

Material	Vendor (Catalog Number)
PCR Plate	Fisher (E951020460)
8 Strip Flat Caps	Fisher (E0030124847)
Molecular Grade Water	Fisher (BP281910)
PCR 2X Master Mix	Promega (M7832)
Nested PCR and ASPE Primers	IDT
ExoSAP-IT	ThermoFisher (78201.1.ML)
dNTP Solution Set	NEB (N0446S)
Biotin-14-dATP	ThermoFisher (19524016)
NEB Taq Polymerase wih 10X Buffer	NEB (M0273S)
Streptavidin, R-Phycoerythrin Conjugate (SAPE)	Thermofisher (S-866)
xTAG 10X Buffer	Luminex (GR001C0060)
Drive Fluid	Luminex (40-50030)
Calibration Kit	Luminex (MPX-CAL-K25)
Performance Verification Kit	Luminex (MPX-PVER-K25)
Microspheres	Luminex
25 mL Reservoir	Fisher (14387070
Amber 5 mL Tube	Axygen (MCT-500-X)
2 mL Tube	Eppendorf (022363352)
15 mL Tube	Eppendorf (0030122151)

Luminex Microspheres

Bead Region	Catalog Number	Anti-TAG Sequence Bound to Each Microsphere (5'-3')	Complementary Primer TAG Sequence (5'-3')	Matching ospC Genotype
12	MTAG-A012	AGTAGAAAGTTGAAATTGATTATG	CATAATCAATTTCAACTTTCTACT	All
14	MTAG-A014	ATTGTGAAAGAAAGAGAAGAAATT	AATTTCTTCTCTTTCTTTCACAAT	A
15	MTAG-A015	GTTGTAAATTGTAGTAAAGAAGTA	TACTTCTTTACTACAATTTACAAC	B
18	MTAG-A018	GTAATTGAATTGAAAGATAAGTGT	ACACTTATCTTTCAATTCAATTAC	D
20	MTAG-A020	AAATTAGTTGAAAGTATGAGAAAG	CTTTCTCATACTTTCAACTAATTT	K
22	MTAG-A022	GATTGATATTTGAATGTTTGTTTG	CAAACAAACATTCAAATATCAATC	I/C
30	MTAG-A030	GTGTTATAGAAGTTAAATGTTAAG	CTTAACATTTAACTTCTATAACAC	M
36	MTAG-A036	TTGTGTAGTTAAGAGTTGTTTAAT	ATTAAACAACTCTTAACTACACAA	E
38	MTAG-A038	AGTAAGTGTTAGATAGTATTGAAT	ATTCAATACTATCTAACACTTACT	T
42	MTAG-A042	ATTTGTTATGATAAATGTGTAGTG	CACTACACATTTATCATAACAAAT	F
44	MTAG-A044	AATGTAAAGTAAAGAAAGTGATGA	TCATCACTTTCTTTACTTTACATT	G
46	MTAG-A046	GTGATTGAATAGTAGATTGTTTAA	TTAAACAATCTACTATTCAATCAC	H
48	MTAG-A048	TATGAATGTTATTGTGTGTTGATT	AATCAACACACAATAACATTCATA	I
52	MTAG-A052	GTAAGATTAGAAGTTAATGAAGAA	TTCTTCATTAACTTCTAATCTTAC	J
54	MTAG-A054	TAGAGAAAGAGAGAATTGTATTAA	TTAATACAATTCTCTCTTTCTCTA	L
56	MTAG-A056	AATTAGAAGTAAGTAGAGTTTAAG	CTTAAACTCTACTTACTTCTAATT	N
61	MTAG-A061	TATTAGAGAGAAATTGTAGAGATT	AATCTCTACAATTTCTCTCTAATA	O
63	MTAG-A063	TTTGTTGTTAAGTATGTGATTTAG	CTAAATCACATACTTAACAACAAA	U
65	MTAG-A065	TGAGTAAGTTTGTATGTTTAAGTA	TACTTAAACATACAAACTTACTCA	V
67	MTAG-A067	TTTGTGTGTTATTGTAATTGAGAT	ATCTCAATTACAATAACACACAAA	W
72	MTAG-A072	AATTGAGAAAGAGATAAATGATAG	CTATCATTTATCTCTTTCTCAATT	E/C

Table 1: Luminex microspheres (beads)

Equipment

1. Thermal cycler
2. Luminex MAGPIX instrument

3. 96-well plate centrifuge
4. Multichannel pipettes

PREPARING SOLUTIONS

1 1X xTAG buffer

1. Add 1 mL of 10X xTAG buffer to 9 mL of molecular grade water. Scale volume up or down as necessary.
2. Store at 4°C until use

2 Bead mix solution (75 beads/µL)

The specific Luminex microspheres (beads) are sold in concentrations of 2.5X10^6 beads/mL. The final concentration of a working bead mix solution is 75 beads/µL. For a 96 well plate, make enough bead mix for 115 samples (~1.2X) to account for pipetting error. Scale volume up or down as necessary.

1. Vortex each bead type (Table 1 from Materials) for at least 20 seconds
2. Add 69 µL of each bead type to an amber 5 mL tube
3. The volume with 21 bead types will be 1449 µL
4. Add 851 µL of 1X xTAG buffer to bring total volume to 2300 µL
5. Store at 4°C until use

3 ASPE primer mix solution (500 nM)

1. Dilute ASPE primers (from IDT) to 200 uM
2. In 2 mL tube, add 947.5 µL of molecular grade water
3. Add 2.5 µL of each ASPE primer (Table 2 below)
4. Vortex and store at -20°C until use

Primer	Sequence (5'-3')
ospC ALL Tag 12	CATAATCAATTTCAACTTTCTACTAGATTAGGCCCTTTAACAGACTCATC
ospC Type A Tag 14	AATTTCTTCTCTTTCTTTCACAATATTGTGATTATTTTCGGTATCC
ospC Type B Tag 15	TACTTCTTTACTACAATTTACAACCTCGTTGCGATTTGCTTCA
ospC Types E/C Tag 72	CTATCATTTATCTCTTTCTCAATTTGCAAGTAAGGTCTCAACTT
ospC Types I/C Tag 22	CAAACAAACATTCAAATATCAATCTCCGTTGTTATCTGCCTCATTATCT
ospC Type D Tag 18	ACACTTATCTTTCAATTCAATTACATGATTATTTAGAGTGCCTAAAGCATTGTTTTGATC
ospC Type E Tag 36	ATTAAACAACTCTTAACTACACAATGTGTTTTTACTCTGATTGGCCTCTAAACCATTATTGCC
ospC Type F Tag 42	CACTACACATTTATCATAACAAATCGCCTGAACGCCTAAACCATTTGCATC
ospC Type G Tag 44	TCATCACTTTCTTTACTTTACATTGGTGTTGTGATTCGCATCAG
ospC Type H Tag 46	TTAAACAATCTACTATTCAATCACGCCCCCATCGTCACCCAAAGTGCCATTTTG
ospC Type I Tag 48	AATCAACACACAATAACATTCATATTTGAAATTAAATATGCTCCTGA
ospC Type J Tag 52	TTCTTCATTAACTTCTAATCTTACTCCGTTTTGACCCACTTCAGC
ospC Type K Tag 20	CTTTCTCATACTTTCAACTAATTTCCCCGCTTCGACAGCTAAACCACCATTTTGTTG
ospC Type L Tag 54	TTAATACAATTCTCTCTTTCTCTAATCGCTACCTAAAGTACCACCTGCTTC
ospC Type M Tag 30	CTTAACATTTAACTTCTATAACACACCGGCATTTAAACCATTTTGGGCTATCAAA
ospC Type N Tag 56	CTTAAACTCTACTTACTTCTAATTGTTTTGCACATCATCTAAACCATTATTATT
ospC Type O Tag 61	AATCTCTACAATTTCTCTCTAATATTGGTTAACTAAGCCATTTGCC
ospC Type T Tag 38	ATTCAATACTATCTAACACTTACTATGGCCTGCATCGACACT
ospC Type U Tag 63	CTAAATCACATACTTAACAACAAACTGCCCTTGCAAGTCCTGT
ospC Type V Tag 65	TACTTAAACATACAAACTTACTCAGAGCCGCTTGAGCAGTTAAACCATTTGCACC
ospC Type W Tag 67	ATCTCAATTACAATAACACACAAATCGTTTCGATTTGCTTCTACACCC

Table 2: ASPE Primers

4 ASPE dNTP Mix (50 µM)

Before making this solution, dilute the dTTP, dCTP, and dGTP (NEB) from 100 mM to 10 mM, and

discard the dATP

1. In 2 mL tube, add 430 µL of molecular grade water
2. Add 2.5 µL of each 10 mM dTTP, dCTP, and dGTP
3. Add 62.5 µL of 0.4 mM biotin-14-dATP
4. Vortex and store at -20°C until use

5 **Streptavidin, R-phycoerythrin conjugate (SAPE) solution**

The SAPE is supplied as 1 mg/mL solution. Dilute SAPE to 10 µg/mL in 1X xTAG buffer. For a 96 well plate, make enough SAPE solution for 116 samples (~1.2X). Scale up or down as necessary.

1. In a 15 mL tube, aliquot 87 µL SAPE into 8613 µL 1X xTAG buffer
2. Total volume is 8700 µL
3. Make fresh immediately before use in step 12 below

NESTED PCR

6 Note: Every batch of samples must have three no template controls (NTCs) included at the nested PCR step. They are used to determine positive *ospC* genotypes after analysis on the MAGPIX instrument, and to confirm that no contamination occurred during the assay.

First round PCR

Master mix preparation

1. Prepare master mix according to Table 4 below for each sample. Scale up depending on the sample size
2. Add master mix components to a tube, vortex, and store on ice

A	B
**Primer**	**Sequence (5'-3')**
ospC1F	ATGAAAAAGAATACATTAAGTGCA
ospC622RC	TTGGACTTTCTGCCACAACA

Table 3: First round nested PCR primers

A	B
**Component**	**Volume (µL)**
Promega PCR Master Mix (2X)	12.5
ospC1F (10 uM)	0.5
ospC622RC (10 uM)	0.5
Molecular grade water	10.5

Table 4: First round PCR master mix

Loading master mix and template DNA

1. Aliquot 24 µL of the master mix into a well for each sample
2. Aliquot 1 µL of template DNA or NTC
3. Seal wells firmly with cap strips
4. Vortex and centrifuge plate at 2500 RPM for 1 minute

5. Load plate into thermal cycler and run program in Table 5 (step 7)

7

Nested PCR Program

A	B	C
**Temperature (°C)**	**Time**	**Cycles**
95	2 minutes	1
95	30 seconds	32
49	30 seconds	32
72	50 seconds	32
72	10 minutes	1
4	Hold	1

Table 5: Nested PCR program

8 Second Round PCR

Master mix preparation

1. Prepare master mix according to Table 7 below for each sample. Scale up depending on the sample size
2. Add master mix components to a tube, vortex, and store on ice

A	B
**Primer**	**Sequence (5'-3')**
OC6(+)24	AAAGAATACATTAAGTGCGATATT
OC602(-)22	GGGCTTGTAAGCTCTTTAACTG

Table 6: Second round nested PCR primers

A	B
**Component**	**Volume µL**
Promega PCR Master Mix (2X)	12.5
OC6(+)24 (10 µM)	0.5
OC602(-)22 (10 µM)	0.5
Molecular grade water	10.0

Table 7: Second round PCR master mix

Loading master mix and template DNA

1. Aliquot 23.5 µL of the master mix into a well for each sample
2. Add 1.5 µL of the DNA template or NTC from the first round PCR into the correct well
3. Seal wells firmly with cap strips
4. Vortex and centrifuge plate at 2500 RPM for 1 minute
5. Load plate into thermal cycler and run program in Table 5 (step 7)

EXOSAP-IT

9 1. Aliquot 7.5 µL of amplified DNA into wells on a new plate
2. Add 3 µL of ExoSAP-IT to each well
3. Seal wells firmly with cap strips
4. Vortex and centrifuge plate at 2500 RPM for 1 minute
5. Load plate into thermal cycler and run program in Table 8

A	B	C
**Temperature (°C)**	**Time (minutes)**	**Cycles**
37	15	1
80	15	1
4	Hold	1

Table 8: ExoSAP-IT program

ALLELE SPECIFIC PRIMER EXTENSION (ASPE)

10 **Master mix preparation**

1. Prepare master mix according to Table 9 below for each sample. Scale up depending on the sample size
2. Add master mix components to a tube, vortex, and store on ice

A	B
**Component**	**Volume (µL)**
Molecular grade water	9.75
10X NEB PCR buffer	2
ASPE primer mix (500 nM)	2
ASPE dNTP mix (50 µM)	1
NEB Taq polymerase (5 U/µL)	0.25

Table 9: ASPE master mix

1. Aliquot 15 µL of the ASPE master mix into wells on a new plate
2. Aliquot 5 µL of the amplicon treated product (from step 9) into the correct wells
3. Seal wells firmly with cap strips
4. Vortex and centrifuge plate at 2500 RPM for 1 minute
5. Load plate into thermal cycler and run program in Table 10

A	B	C
**Temperature (°C)**	**Time**	**Cycles**
95	2 minutes	1
95	30 seconds	35
56	30 seconds	35
68	30 seconds	35
68	5 minutes	1
4	Hold	1

Table 10: ASPE program

BEAD HYBRIDIZATION

11

1. Vortex bead mix for at least 30 seconds
2. Aliquot 20 µL of bead mix into wells on a new plate
3. Aliquot 3.5 µL of the ASPE product (from step 10) into the correct wells
4. Gently pipette up and down several times to mix
5. Seal wells firmly with cap strips
6. Load plate into thermal cycler and run program in Table 11

A	B	C
**Temperature (°C)**	**Time (minutes)**	**Cycles**
96	2	1
37	30	1

Table 11: Bead hybridization program

ANALYSIS ON MAGPIX

12 **Addition of SAPE (reporter solution)**

1. Invert SAPE (10 µg/mL) several times and pour into 25 mL reservoir
2. Aliquot 75 µL SAPE solution to every well
3. Gently pipette up and down several times to mix
4. Transfer samples to pre-warmed heater block (37°C) on MAGPIX instrument
5. Incubate at 37°C for 15 minutes

13 **Analyze samples on MAGPIX**

Please refer to Luminex technical support and the MAGPIX manual for detailed instructions on how to create a protocol, run a batch of samples, and MAGPIX maintenance. Specific settings for our *ospC* genotyping protocol are listed below.

1. 70 µL sample volume
2. Sample wash "on"
3. Heater set at 37°C
4. 50 bead count minimum

DATA ANALYSIS

14 At the end of the run, a .csv file will be created containing the protocol/run information and the raw median fluorescent intensity (MFI) results for each sample

1. Open the .csv file in Microsoft Excel
2. Copy the "DataType: Median" results for the three NTCs and every sample to a new sheet in the workbook
3. Using the three NTCs, calculate the average background MFI value for every *ospC* genotype (analyte)
4. Add three standard deviations to the averages. These values (NTC values) are specific for each genotype. NTC values are determined for each genotype, since background can vary slightly between genotypes.
5. For individual samples, raw *ospC* genotype MFI values that have a ratio to NTC value (RNTC) of ≥ 3 are considered positive

Example

1. For *ospC* genotype A the MFI values in the three NTCs were (100, 125, and 150)
2. The average (125) plus three standard deviations (20.4) is 186 (NTC value)
3. The raw MFI value for *ospC* genotype A in sample "X" is 800
4. Sample "X" is positive for *ospC* genotype A since the RNTC value is 4.3
