# Supplementary material for: A Borrelia burgdorferi outer surface protein C (OspC) genotyping method using Luminex technology
Source: PLoS One. 2022 Jun 1;17(6):e0269266. doi: 10.1371/journal.pone.0269266 (PMC9159548; doi:10.1371/journal.pone.0269266)

Table 1. Qiu et al. K probe and modified K primer. Nucleotide differences are highlighted in bold.

| **Description** | **Sequence (5’-3’)** |
| --- | --- |
| Qiu et al. K Probe | CCCCGCTTCG**CT**A**C**CTAAACCA**G**CATTTTGTTG |
| Modified K Primer (used in this assay) | CCCCGCTTCG**AC**A**G**CTAAACCA**C**CATTTTGTTG |

Figure 1. Alignment of *ospC* K reference sequence with Qiu et al. K probe and modified K primer.


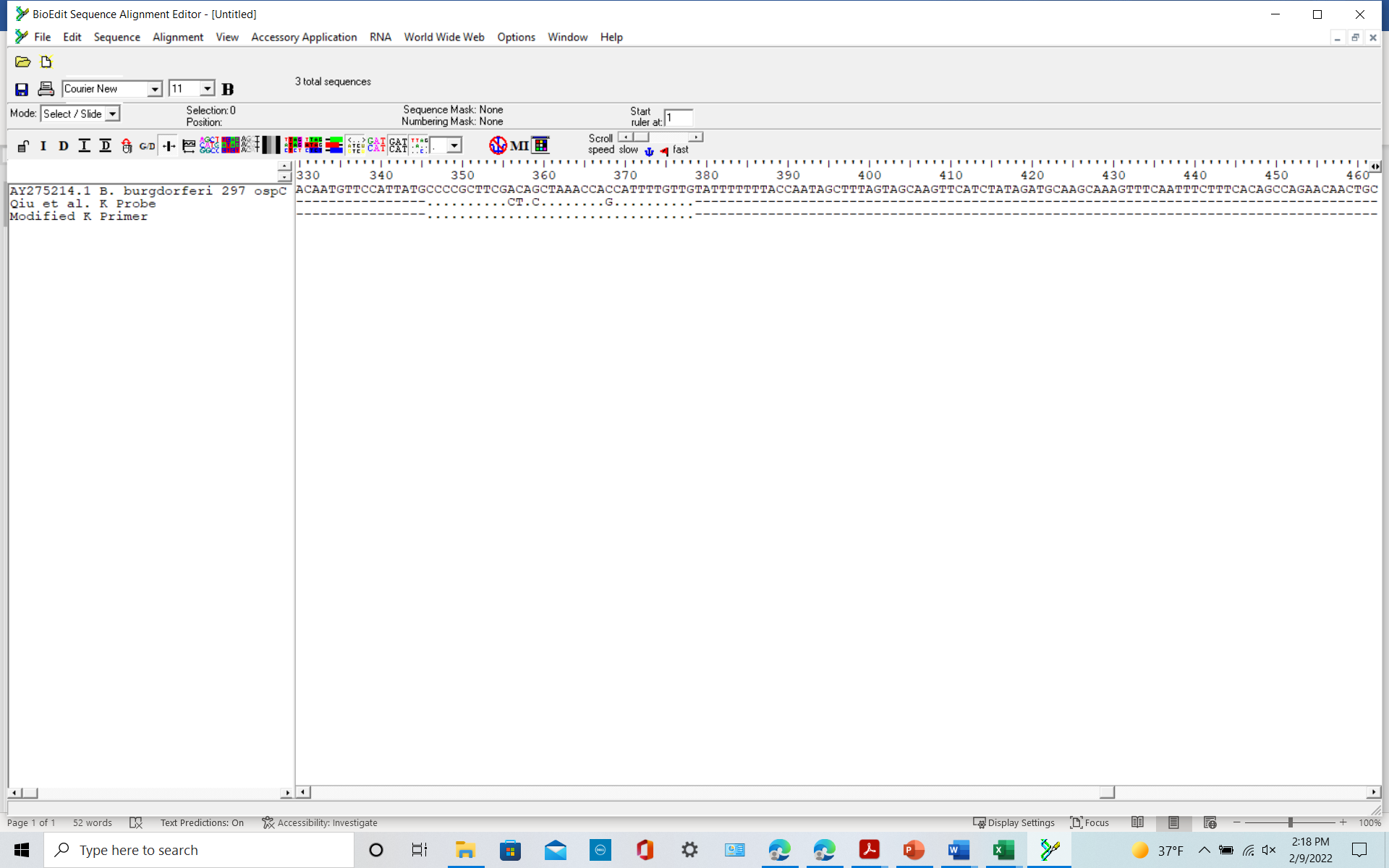

Supplement: S2 File — The Qiu et al. K probe and our modified K primer sequences are shown in Table 1, nucleotide differences are highlighted in bold. Fig 1 shows an alignment of the Qiu et al. K probe and our modified K primer with an ospC K reference sequence (Genbank accession number AY275214). (DOCX) [file pone.0269266.s002.docx]
