# Supplementary material for: A Borrelia burgdorferi outer surface protein C (OspC) genotyping method using Luminex technology
Source: PLoS One. 2022 Jun 1;17(6):e0269266. doi: 10.1371/journal.pone.0269266 (PMC9159548; doi:10.1371/journal.pone.0269266)
Supplement: S3 File — The nested PCR first round primer hybridization sites are highlighted in blue and the second round primer hybridization sites are highlighted in red. Overlapping regions for the first and second round forward primers are highlighted in purple. The hybridization site for the ASPE “ALL” primer is highlighted in green. The ASPE primer hybridization sites for each ospC genotype are highlighted in yellow. The ospC A-U reference sequences were obtained from Di et al. [11]. The ospC V and W accession numbers are FJ649656 and FJ649657, respectively. (DOCX) [file pone.0269266.s003.docx]

10 20 30 40 50 60 70 80 90 100

....|....|....|....|....|....|....|....|....|....|....|....|....|....|....|....|....|....|....|....|

**ospC A** **ATGAAAAAGAATACATTAAGTGCAATATTAATGACTTTATTTTTATTTATATCTTGTAATAATTCAGGGAAAGATGGGAA---TACATCTGCAAATTCTG**

**ospC B** **................................................................................---.................**

**ospC C** **....................................................................A...........---.G...............**

**ospC D** **....................................................................A...........---.................**

**ospC E** **....................................................................A...........---.G...............**

**ospC F** **....................................................................A...........---.................**

**ospC G** **....................................................................A...........---.G.....A.........**

**ospC H** **................................................................................---.G...............**

**ospC I** **................................................................................---.................**

**ospC J** **....................................................................A...........---.................**

**ospC K** **....................................................................A...........---.................**

**ospC L** **.......................G.....G......................................A...........---.G......T........**

**ospC M** **................................................................................---.................**

**ospC N** **................................................................................---.G.....A.........**

**ospC O** **................................................................................---.................**

**ospC T** **.............................G......................................A...........---.G......T........**

**ospC U** **....................................................................A...........---.G...............**

**ospC V** **------------------------------......................................A...........CGC.G.....A.T...C...**

**ospC W** **---------------------------------------------.......................A...........---.................**

110 120 130 140 150 160 170 180 190 200

....|....|....|....|....|....|....|....|....|....|....|....|....|....|....|....|....|....|....|....|

**ospC A** **CTGATGAGTCTGTTAAAGGGCCTAATCTTACAGAAATAAGTAAAAAAATTACGGATTCTAATGCGGTTTTACTTGCTGTGAAAGAGGTTGAAGCGTTGCT**

**ospC B** **....................................................................................................**

**ospC C** **....................................................A..A.....C..A...G.T..C..C........A.....GA.C..A..**

**ospC D** **.............................................................................................T......**

**ospC E** **....................................................A..A.....C..A...G.T..G..C........A.....GA.C..A..**

**ospC F** **....................................................A..A.....C..A...G.T..G.....A.....AA.....A.T.....**

**ospC G** **.C...........................G......................A..A........A...G....G.....A.....A....CG........**

**ospC H** **....................................................A..A.....C..A...G....G.....A.....A......A.C.....**

**ospC I** **....................................................A..A.....C..A...G.T..C..C........A......A.T.....**

**ospC J** **....................................................A..A.....C..A...G.T..G...........AA.....A.T.....**

**ospC K** **....................................................A..A.....C..A...G.T..G...........AA.....A.T.....**

**ospC L** **.............................GT.....................C...............G.TA....A........A......A.T.....**

**ospC M** **....................................................A..A.....C..A...G.T..C..C........A......A.TC....**

**ospC N** **.C..................................................A..A........A...G....G.....A.....A....CG........**

**ospC O** **....................................................A..A.....C..A...G.T..G.....A.....A.....G..T.....**

**ospC T** **....................................................A..A.....C..A...G.T..C..C........A......A.TC....**

**ospC U** **.............................G......................A..A.....C..A...G.T..G.....A.....A.....G..T.....**

**ospC V** **....................................................A...........A...G.T..C..C........A......A.T.....**

**ospC W** **....................................................A..A.....C..A...G.T..G.....A.....AA.....A.T.....**

210 220 230 240 250 260 270 280 290 300

....|....|....|....|....|....|....|....|....|....|....|....|....|....|....|....|....|....|....|....|

**ospC A** **GTCATCTATAGATGAAATTGCTGCTAAAGCTATTGGTAAAAAAATACACCAAAATAATGGTTTGGATACCGAAAATAATCACAATGGATCATTGTTAGCG**

**ospC B** **...............GC.....---.....................A.AA.CG..GG.A....A....AT...GCA....G...C.AG...........A**

**ospC C** **TG..............C.....---.....................A.AA.CG..GT.A....A....AT..GGCAG..A....C........AA....A**

**ospC D** **................C.....AAG.....................G.T.....C....C...A.GC..TCT.G.......T..C...........G...**

**ospC E** **TG..............C.....A.C.....................GG.---...........A..GG..A.TC.G.G.A.A..CAC..........T.A**

**ospC F** **T...............C.....A..............C........G.TGC....GG.---..A.GCGTTC.GGCG.....A..C..............A**

**ospC G** **T...............C.....---.....................G.G......GG.---....G.G.T..TGCG........CACC...........A**

**ospC H** **TG........A.CC..C.....A---....................G.T......GGCAC.....G.GA...TGGGGGC..A............C....A**

**ospC I** **TA.............GC.....---.....................A.AA.CG..GT.A....A....AT..GGCAG.......C........AA..T.A**

**ospC J** **TG..............C.....A.......................G.TA.C...GC........G.G.T...GTGGG...A..C.........C....A**

**ospC K** **TG..............C.....A.........................A......GG......A.C.GT....GCGGGG..T......A..........A**

**ospC L** **TGT............GC.....A---....................G.AGC.GG.GG.AC...A.G..G...TGGGGCA.....C........AC....A**

**ospC M** **TG..............G.....AAG...........G..TTTG...GC..---.A........AA..G...GCGCA.....A..C...............**

**ospC N** **T..............GC.....---.....................A.TA.T...GG.---..A...GAT.TGC.A..CTT...C.C......A..G..A**

**ospC O** **T..............GC.....---...............G.....GGGGC....GGC---..A.T..A.C..GCA.....T..C.T............A**

**ospC T** **TG..............C.....AA.............C........G.T...............AG.GT...TGCAGGC..T......C..........A**

**ospC U** **TG..................G.AG.................G......AGCG...GG.TTACA...CTTGC..G---GG..G..C..............A**

**ospC V** **TA.............GC.....---............C........GGTGC....GG.---..AAC.G.TC..GCGGC...A..............G..A**

**ospC W** **T...............C.....A.........................G.......G...A....G.GTA...GCA....GA..C.A.......C....A**

310 320 330 340 350 360 370 380 390 400

....|....|....|....|....|....|....|....|....|....|....|....|....|....|....|....|....|....|....|....|

**ospC A** **GGAGCTTATGCAATATCAACCCTAATAAAACAAAAA---------TTAGATGGATTGAAAAAT---GAAGGATTAAAGGAAAAAATTGATGCGGCTAAGA**

**ospC B** **.........A...........T......C.......---------...AG.AA...A..CGGATCA.....T...........G....CC..A.......**

**ospC C** **.........A...........T......C.......---------...AG.AA...A..CGGATCA.....T...........G....CC.AA.......**

**ospC D** **...........T......G.T.......C.G.....---------...AG.TC.A.A...G..TCA.G..A...G....C.G.......AAA........**

**ospC E** **.................TGA.......GC.G.....---------...A...T..........---....A............G......A.A......C**

**ospC F** **..T..C......................C.......---------...AG..C...A..---.TCA....AC.....A......G...CAAA..T.....**

**ospC G** **......C...A.........................---------..................---...........TA..G.......A..A.......**

**ospC H** **..............C.....TG......T.G.....---------..GAGCAC...A......GTA....A......A.........ACAAA.......G**

**ospC I** **.....A...TT...T.....AT......C.A.....---------A..AG..C.A.A...G..TCA.G..A...G....C.G.......AAA........**

**ospC J** **..............C.....TG......T.G.....---------..GAGCAC...A......GTA....A......A.........ACAAA.......G**

**ospC K** **..T......A.........AA.......C.......---------..................TCA...AA..................AAAT.......**

**ospC L** **..G..C...AA....G.....GA.....C.GC...T---------...AGCAA...A...GC.TCA....AC.....A.........ACAAA.......G**

**ospC M** **.....C..C.T................GC.G.....---------..................TCA....A..................A.AT.....A.**

**ospC N** **......C..A.........AAT..G...C.G.....---------...AGCAA..........TCA...........A...........G.AC..C..A.**

**ospC O** **.....C....A.........T.......C.G.....---------...AG.AA...A..CGG.TCA.................G....CC..A.......**

**ospC T** **.....C..C.........G.T.......C.G.....---------...A.......A.C..TCTCA....A............G.....AAA......A.**

**ospC U** **.....C........T....ATT......C.......ATAAATGTA...A.....C........TCA....A...G..A..G......A.C.A.......G**

**ospC V** **.....C............T..T......C.G.....---------...A..AA...T...G..TTA..............G......A...AT..C....**

**ospC W** **.......G..T.................C.G.....---------...AG.AA...A.GCGGATCA.....T.................A..........**

410 420 430 440 450 460 470 480 490 500

....|....|....|....|....|....|....|....|....|....|....|....|....|....|....|....|....|....|....|....|

**ospC A** **AATGTTCTGAAACATTTACTAATAAATTAAAAGAAAAACACACAGATCTTGGTAAAG------AAGGTGTTACTGATGCTGATGCAAAAGAAGCCATTTT**

**ospC B** **....C......GAG....G..C....C.......T..T..TG..C.G.......T.C------.G..C..........AAA........A....T.....**

**ospC C** **....C......GA.........A.................................A------...A..C........T.C.............T.....**

**ospC D** **............GC........A...C..TC...T..T..AG....G.......T..------.GAA..C........A.A........A.G..T.....**

**ospC E** **........AC.GA.............C.....AGTG....TG...TG......CTG.------.CAA.C.........A.A.....C..AG...T.....**

**ospC F** **.......C...GAC..C.....C..G......A.TGGTA.TG..C.G.....CTT..------CT.C..C........A.A.........CG..G.....**

**ospC G** **.........C.G..........A...C..GC...T.GTA.TG..........AGTG.CTGCTGGTAA..C........A.A........AG...T.....**

**ospC H** **.T.....C....AG...G..GGC.........A.TG.....G..AG..........A------...A..C........A..........A....T.....**

**ospC I** **...........GA.......GC...........GTG.....................------....C..........A.A........A..........**

**ospC J** **.T..........A...C.....A...........T.GT...G....G.........A------...A..CC.G.....A..........A....T.....**

**ospC K** **...........GAT........A...C..G...G.G....TG.GC.A.....A.TT.------..AA...........AGA........A....T.....**

**ospC L** **..........G.A.......G.....C.....AGTG..A.TGT..CG.....C...C------.G.A..C..G.....A..........A....T.....**

**ospC M** **.....AACA..G........G.....C.....AGT.GT..TG.G..A..C....T..CGAATGG..C..C..........A.........CG..T.....**

**ospC N** **..........TGAT........A...C..C..TCT.GC..TG..C.G......GTT.CTGGTGGT.C.AC........AA..G..T...A....T.....**

**ospC O** **....C......GAG....G..CA...C.....AGT.GTA.TG..C.G...AA.C.G.------CCAA..C..A.......A.........CG..T.....**

**ospC T** **........AC.GGC.....C......C.....AGTGGT..TG....G......CC..TGGGTGGGAA..C........AAA........C....T.....**

**ospC U** **GC..C.......A.........A...C..TC.....GT..TG.....A......T.C------...CC.C..........A..........T..T.....**

**ospC V** **...........G.G...................GGG.G..GG..ACG.....CC..C------..AA..C..G.....A..........A.G..T.....**

**ospC W** **.T.....A...CA.........A...........T.GT..TGG...A......GT.C------.GAA...........A.A........A..........**

510 520 530 540 550 560 570 580 590 600

....|....|....|....|....|....|....|....|....|....|....|....|....|....|....|....|....|....|....|....|

**ospC A** **AAAAACAAATGGTAC---TAAAACTAAAGGTGCTGAAGAACTTGGAAAATTATTTGAATCAGTAGAGGTCTTGTCAAAAGCAGCTAAAGAGATGCTTGCT**

**ospC B** **....G......CAG.GGG....GA...G..C.T...........A...G..G.CC.G....T....AAG...A...........................**

**ospC C** **...............---....GA...G..C....C...G....A.....................AAA....G......................AT.C**

**ospC D** **.......C..AA.G.---....GAC..G................T...G....C...........CA.G...A.T..........C...CC..A..G...**

**ospC E** **.....A.C...CA.A---....GA...G.......C........A...G......A..G.G.....AAA...A............C....C.CAT.AAAA**

**ospC F** **............G..---...CGA...G..C...A........AA.G.TC...C...T........AAG....GT..........C...TA.....AA..**

**ospC G** **.......C....GCA---.G.GGAC..G....G.A.......CAA.G.G....C....G.....A.AAG....CTT........GC...CAGCAT.A...**

**ospC H** **...G...C....A.A---..CCGA...G..C...A........AA.G.TC...C...T........AAG....GT......G..............AA..**

**ospC I** **..........AA.GA---.........G..C.....T.......A...G...............A.AAA............................A..**

**ospC J** **..........CAAG.---...CGA...G......A........AA.G.G.........G.......AAG............G..............AAAC**

**ospC K** **..T....G...CAG.---....GA...G..C....C...G....A...GC.....A..G.......AAA....G..........................**

**ospC L** **.......C..AA.GA---..T......G......A........AA.G.G....CA........G...AC.....T.........................**

**ospC M** **...............---....GA...G......C....G....A...G...............A.AAA................C....A.CA..AAA.**

**ospC N** **..G......C.CA.T---....GA...G..C..A..T.......A...G......A..........AAG...AG...........C....CGCA..A..C**

**ospC O** **.......C..AA...---....GAC..G................T...G...GC...........CA.G.....T.....T...GC....A.....AAAC**

**ospC T** **......TC....A.A---.GTT.....G..C...A....G...AA.G.T....CC........G..A.C...AG...........C...CA.....AA..**

**ospC U** **..........CC...---.........G................AT..G......A..G.G..G..AAA............G..................**

**ospC V** **.......C....A..---.CCCGA...G.....CA....G...AA.G.T....CC..........CA.G.....T.....T..-----------------**

**ospC W** **.......C..AA.GA---.........G..C.....T.......A...G......A..G......................................A..**

610 620 630 640 650

....|....|....|....|....|....|....|....|....|....|....|..

**ospC A** **AATTCAGTTAAAGAGCTTACAAGCCCTGTTGTGGCAGAAAGTCCAAAAAAACCTTAA**

**ospC B** **..................................T......................**

**ospC C** **..................................................C......**

**ospC D** **.......................T.................................**

**ospC E** **...G.T.................T...A.............................**

**ospC F** **.......................T.................................**

**ospC G** **.........C.............T................C................**

**ospC H** **.......................T.................................**

**ospC I** **.........................................................**

**ospC J** **..G....................T...A......................C......**

**ospC K** **.......................T...A......................C......**

**ospC L** **..............A........T.................................**

**ospC M** **..............A........T................A................**

**ospC N** **...........C.........G.T................C................**

**ospC O** **..............A........T................A................**

**ospC T** **.......................T................C................**

**ospC U** **..............A........T.................................**

**ospC V** **---------------------------------------------------------**

**ospC W** **...G..........-------------------------------------------**
