# Supplementary material for: A Borrelia burgdorferi outer surface protein C (OspC) genotyping method using Luminex technology
Source: PLoS One. 2022 Jun 1;17(6):e0269266. doi: 10.1371/journal.pone.0269266 (PMC9159548; doi:10.1371/journal.pone.0269266)
Supplement: S1 Table — For LOG, a representative run of 90 samples was chosen to leave room on the 96-well plate for negative and positive controls, as detailed in the step-by-step protocol. (DOCX) [file pone.0269266.s006.docx]

|  | **LOG** | **RLB** |
| --- | --- | --- |
| Number of samples analyzed per run | 90 | 40 |
| Time per run (hours) | 5 | 4 |
| Number of samples per hour | 18 | 10 |
