# Supplementary material for: A Borrelia burgdorferi outer surface protein C (OspC) genotyping method using Luminex technology
Source: PLoS One. 2022 Jun 1;17(6):e0269266. doi: 10.1371/journal.pone.0269266 (PMC9159548; doi:10.1371/journal.pone.0269266)
Supplement: S2 Table — (DOCX) [file pone.0269266.s007.docx]

| **Nested PCR Reaction** | **Primer** | **Sequence (5’-3’)** | **Reference** |
| --- | --- | --- | --- |
| First round | ospC1F | ATGAAAAAGAATACATTAAGTGCA | This study |
|  | ospC622RC | TTGGACTTTCTGCCACAACA | This study |
| Second round | OC6(+)24 | AAAGAATACATTAAGTGCGATATT | [3] |
|  | OC602(-)22 | GGGCTTGTAAGCTCTTTAACTG | [3] |
