# Supplementary material for: A Borrelia burgdorferi outer surface protein C (OspC) genotyping method using Luminex technology
Source: PLoS One. 2022 Jun 1;17(6):e0269266. doi: 10.1371/journal.pone.0269266 (PMC9159548; doi:10.1371/journal.pone.0269266)
Supplement: S3 Table — ospC genotype standards (ospC gBlocks and a K plasmid stock) were amplified, diluted to approximately 5 ng/ul in samples representing single and mixed B. burgdorferi ospC genotype infections, and then analyzed using LOG. As described in the step-by-step protocol, a ratio to NTC value of ≥ 3 was considered positive. (DOCX) [file pone.0269266.s008.docx]

| **Amplified ospC Genotype(s)** | **Genotype(s) Detected** | **MFI** | **Ratio to NTC Value** |
| --- | --- | --- | --- |
| A | A | 1254 | 8.2 |
| B | B | 3188 | 18.6 |
| C | C | E/C (2361), I/C (3824) | E/C (15.5), I/C (22.8) |
| D | D | 3350 | 18.4 |
| E | E | E (2984), E/C (2317) | E (17.0), E/C (15.2) |
| F | F | 6298 | 39.8 |
| G | G | 3866 | 24.1 |
| H | H | 3401 | 19.6 |
| I | I | I (3203), I/C (3986) | I (20.8), I/C (23.7) |
| J | J | 4989 | 31.0 |
| K | K | 4648 | 22.8 |
| L | L | 3260 | 19.6 |
| M | M | 4578 | 28.8 |
| N | N | 1714 | 10.0 |
| O | O | 4612 | 28.1 |
| T | T | 3879 | 21.3 |
| U | U | 4063 | 23.4 |
| V | V | 5239 | 28.8 |
| W | W | 3420 | 18.5 |
| ABC | ABC | A (1570), B (3259), E/C (2387), I/C (4196) | A (10.3), B (19.0), E/C (15.7), I/C (25.0) |
| EFN | EFN | E (3595), E/C (2194), F (6990),  N (1669) | E (20.4), E/C (14.4), F (44.1),  N (9.7) |
| DGHJ | DGHJ | D (3822), G (4542), H (3819),  J (5923) | D (21.0), G (28.3), H (22.0),  J (36.7) |
| IOT | IOT | I (3432), I/C (4945), O (5475),  T (4379) | I (22.3), I/C (29.4), O (33.3),  T (24.0) |
| KLM | KLM | K (6208), L (4030), M (5177) | K (30.5), L (24.2), M (32.6) |
| UVW | UVW | U (4328), V (6332), W (4092) | U (24.9), V (34.8), W (22.1) |
