# Supplementary material for: A Borrelia burgdorferi outer surface protein C (OspC) genotyping method using Luminex technology
Source: PLoS One. 2022 Jun 1;17(6):e0269266. doi: 10.1371/journal.pone.0269266 (PMC9159548; doi:10.1371/journal.pone.0269266)
Supplement: Supplementary file 8 [file pone.0269266.s005.docx]

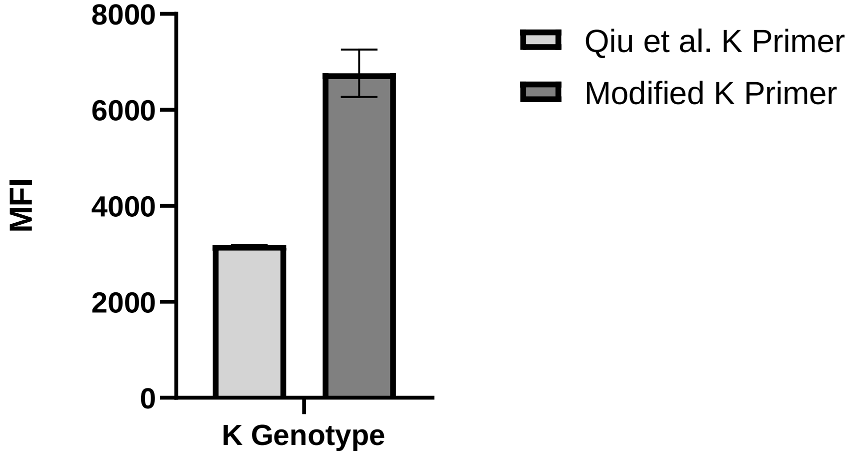

MFI
8000
6000
4000
2000
0
K Genotype
Qiu et al. K Primer
Modified K Primer
